# Whole Genome Sequence of a Turkish Individual

**DOI:** 10.1371/journal.pone.0085233

**Published:** 2014-01-09

**Authors:** Haluk Dogan, Handan Can, Hasan H. Otu

**Affiliations:** Department of Genetics and Bioengineering, Istanbul Bilgi University, Istanbul, Turkey; Yale University, United States of America

## Abstract

Although whole human genome sequencing can be done with readily available technical and financial resources, the need for detailed analyses of genomes of certain populations still exists. Here we present, for the first time, sequencing and analysis of a Turkish human genome. We have performed 35x coverage using paired-end sequencing, where over 95% of sequencing reads are mapped to the reference genome covering more than 99% of the bases. The assembly of unmapped reads rendered 11,654 contigs, 2,168 of which did not reveal any homology to known sequences, resulting in ∼1 Mbp of unmapped sequence. Single nucleotide polymorphism (SNP) discovery resulted in 3,537,794 SNP calls with 29,184 SNPs identified in coding regions, where 106 were nonsense and 259 were categorized as having a high-impact effect. The homo/hetero zygosity (1,415,123∶2,122,671 or 1∶1.5) and transition/transversion ratios (2,383,204∶1,154,590 or 2.06∶1) were within expected limits. Of the identified SNPs, 480,396 were potentially novel with 2,925 in coding regions, including 48 nonsense and 95 high-impact SNPs. Functional analysis of novel high-impact SNPs revealed various interaction networks, notably involving hereditary and neurological disorders or diseases. Assembly results indicated 713,640 indels (1∶1.09 insertion/deletion ratio), ranging from −52 bp to 34 bp in length and causing about 180 codon insertion/deletions and 246 frame shifts. Using paired-end- and read-depth-based methods, we discovered 9,109 structural variants and compared our variant findings with other populations. Our results suggest that whole genome sequencing is a valuable tool for understanding variations in the human genome across different populations. Detailed analyses of genomes of diverse origins greatly benefits research in genetics and medicine and should be conducted on a larger scale.

## Introduction

Following the publication of two draft sequences [1], [2], a highly accurate and nearly complete assembly of the human genome was published in 2004 [3]. In parallel with the low-cost/high-throughput advances in DNA sequencing technology, human whole genome sequencing (WGS) is being performed worldwide at an increasing pace. Individual WGS began to surface with Venter's and Watson's genomes [4], [5], and this approach was quickly adapted to individuals from diverse ethnic backgrounds [6]. Understanding DNA sequence variation sheds light on the relationship between genotype and phenotype, and WGS has proven to be a powerful tool. The 1000 Genomes Project, for example, has performed 185 human WGSs from four populations and discovered about 20,000 novel structural variants in its Pilot Phase [7]. In Phase I of the project, the number of sequenced individuals increased to 1,092 covering 14 populations and identifying 38M single nucleotide polymorphisms (SNPs), 1.4 M indels and over 14 K larger deletions [8].

Efforts (other than WGS) that target discovery of human genome variations also exist, such as the HapMap project [9]. Latest HapMap results cover 1,184 individuals from 11 populations and involve genotyping of common SNPs and sequencing of relatively small regions (∼100 Kbp). Overall, HapMap and similar consortiums have catalogued over 10 million SNPs, 3 million indels, and associated linkage-disequilibrium patterns. This ongoing process of identifying genomic variants has paved the way for genome-wide association studies over the past few years, and disease susceptibility has been found to be associated with these variants for over a thousand regions so far. This accumulated knowledge in the post-genomic era is opening new frontiers in medicine and public health using a personalized approach, and WGS is becoming the method of choice with its ability to construct a nearly complete picture of identifying structural variations.

Despite the increasing use of human WGS for both research and clinical purposes, there remain two areas that require further attention: i) there are populations for which WGS or SNP discovery efforts have not been done; ii) very few of the human WGS performed so far provide high-coverage sequencing results with detailed analysis. Out of the 185 individuals for whom WGS has been performed in the 1000 Genomes Project's Pilot Phase, only six were subjected to high-coverage sequencing (∼42x) while the remaining individuals were subjected to low-coverage sequencing (2–6x). All of the individuals studied in Phase I of the project were analyzed using low-coverage sequencing (2–6x).

There have been various efforts to perform high coverage WGS of different populations with detailed analysis [10], [11], [12], [13], [14], [15]. In order to provide a better and more complete picture of human genome variations, we believe more individuals from diverse populations need to be sequenced and analyzed at a sufficiently detailed level. Therefore, in this paper, we present a high-coverage WGS of a Turkish individual and the results of the associated analysis.

Turkey, the most populous well-defined region inhabited by Turks, is an interesting geographical region as it lies at the crossroads between Europe and Asia. Historically, migration from Central Asia and ancestral contribution to regions surrounding Anatolia, such as the Balkans, Middle East, Caucasian and Caspian regions, has positioned the Turkish population as an interesting genetic resource that requires further detailed analysis. Certain important diseases, such as hemoglobinopathies (e.g. sickle-cell disease), thalassemias, and Behcet's disease, are highly prevalent in the Turkish population; and diseases exist where the Turkish population does not exhibit the variant believed to be the cause [16], [17], [18]. The current study provides a baseline for high-throughput/wide-spectrum analysis of genome variations in the Turkish population, which may lead to a better understanding of the relationship between the genotype and the phenotype through comparative analysis.

## Materials and Methods

### Ethics Statement

This study is approved by the Committee on Ethics in Research on Humans of Istanbul Bilgi University.

### Individual Selection, DNA Isolation, and Genotyping

The genomic DNA (gDNA) used in this study came from a healthy male individual, who was anonymous and was reported to come from Turkish ethnicity for at least three generations. Informed consent was obtained prior to the collection of the blood sample from which gDNA was isolated using a QIAamp DNA blood kit (Qiagen®). The individual gave written consent to the publication of his genome sequence. A quality control inspection and rough quantitation of the gDNA sample was performed by agarose gel electrophoresis and UV-induced ethidium bromide fluorescence (Figure S1). The quality of the sample on the gel was visually compared to New England BioLabs 2-Log DNA Ladder molecular weight size marker. The sample was of acceptable quality for continued processing. The individual was genotyped using Illumina Human CytoSNP-12 V2.1 (Illumina®) SNP chip following the manufacturer's instructions.

### Library Preparation and Sequencing

The genomic DNA sample was used to generate a paired-end library suitable for the HiSeq sequencing platform (Illumina®) prepared using the TrueSeq DNA Sample Preparation kit, following the manufacturer's instructions. Quality control analysis of the library using an Agilent 2100 Bioanalyzer indicated that the library was of acceptable quality, containing the expected fragment size and yield, for continued sample processing (Figure S2). The library generated was used in the cBot System for cluster generation in three flow cell lanes. The flow cell containing amplified clusters was sequenced using 2×101 base pair paired-end sequencing on a Hi-Seq 2000. Bad quality reads were eliminated from the final output of the sequencing machine. In brief, for each cluster, a “chastity” score was calculated, which is the Highest_Intensity/(Highest_Intensity + Next_Highest_Intensity) for a base call in the first 25 cycles. A cluster was retained if it contained at most one base call instance where the chastity parameter was less than the threshold. Remaining reads were further trimmed and filtered using Trimmomatic [19] where reads with a high quality (average Q≥20) score and a minimum length of 36 (after trimming) were kept.

Sanger sequencing was used for validation of 20 small indels identified by computational analysis of the whole genome sequence data. DNA was isolated using a Dual DNA isolation kit (GenedireX Inc., Taiwan). Twenty regions were amplified from 50 ng genomic DNA with 10 pmol of forward and reverse primer pairs. Polymerase chain reaction (PCR) was performed using the following cycling profile: initial denaturation at 95°C for 5 min. followed by 10 cycles of 95°C for 30 s, 63°C for 30 s, and 72°C for 30 s; 25 cycles of 95°C for 30 s, 56°C for 30 s, and 72°C for 30 s; and a final extension step at 12°C for 5 min. Amplicons were purified using GenedireX PCR Clean-Up kit (GenedireX Inc., Taiwan) and quantified. ABI 3100 DNA analyzer and ABI BigDye Terminator cycle sequencing was used for Sanger sequencing.

### Mapping and *De Novo* Assembly

The reference genome used in this study was NCBI human reference genome build 37.1 (GRCh37/hg19 assembly). We adopted two workflows for read mapping. First, we used the vendor supplied Eland and Casava (v1.8) pipeline using recommended settings with a variants covariance cut-off value of 3. Alternatively, we used the Burrows-Wheeler Alignment tool (BWA v0.6.2) for mapping reads to the reference genome [20]. The vendor-supplied method of mapping was used only for SNP calling in the respective pipeline. The mapping results provided in the manuscript are based on the BWA approach. In the application of the BWA tool, we used a minimum seed length of 20 (with a maximum seed distance of 2), an output alignment score cut-off of 30, a maximum edit distance of 0.04, and a maximum insert size of 500. Specifically, we used the *bwasw* algorithm to index the database and generated suffix array indices for two ends in a paired-end read separately. We then combined the two results with the *sampe* algorithm to produce the final sequence alignment/map (SAM) file. BWA analysis results were investigated with SAMStat v1.08 [21] to determine the quality and statistics associated with the mapping step. Unmapped reads were assembled using the iterative De Bruijn Graph De Novo Assembler (IDBA v1.1.0) where the minimum seed length for overlapping nucleotides was set to be 25 [22]. We required at least five pair-end connections to join two contigs and a minimum contig length of 100. The resulting contigs were analyzed using BLAST v2.2.26 [23] on the NCBI's RefSeq genomic database using an E-value cut-off of 10^−10^.

### SNP/Indel and CNV/SV Identification

SNP identification was done using the vendor-supplied Eland-Casava pipeline with the recommended settings and The Genome Analysis Toolkit (GATK v2.2) [24] applied on the BWA output. GATK has also been used in indel identification. Prior to variant discovery, reads were subjected to local realignment, coordinate sort, quality recalibration, and duplicate removal. In the GATK analysis, we used a minimum confidence score threshold of Q30 with default parameters. Annotation of the discovered SNPs/indels and their potential effects were analyzed using snpEff v3.1 [25]. During the SNP/indel discovery and analysis phases, we adopted NCBI's dbSNP build 135. CNV/SV events were discovered using read-depth-based CNVnator and paired-end-based CLEVER algorithms [26], [27]. In the CLEVER approach, we used a maximum insert length of 50,000 and a maximum allowed coverage of 200. In the CNVnator analysis, we used a bin (window) size of 100 with default parameters. CNV/SV calls using the SNP chip data were obtained by QuantiSNP with default parameters [28].

### Functional Analysis

We used Ingenuity Software Knowledge Base (IKB), (Redwood City, CA) for the functional interaction analysis of the genes affected by high-impact, novel SNPs. IKB uses interactions between genes and/or gene products based on manual curation of scientific literature providing a robust interaction database. Once a gene list of interest is identified, IKB uses known interactions between these genes to build an interaction network. The final network includes genes that are not in the input list but are highly connected to the genes in the input list. This feature enables the investigation of new modules of interactions that are not covered by existing canonical pathways. The results are also analyzed in terms of drugs, small metabolites, functions, and diseases that are overlaid on the resulting network. To this end, an annotation of the resulting interaction network is achieved where functional entities involved in the network are underlined.

## Results

### Individual Selection, DNA Isolation, and Genotyping

**Figure 1:**
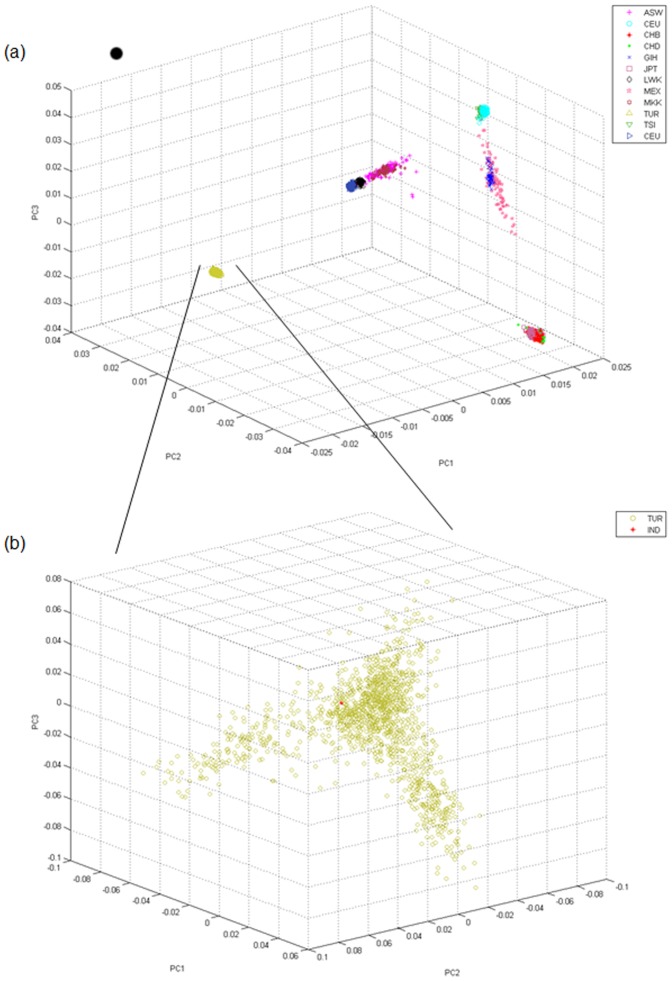
Position of the Turkish population and the sequenced individual based on Principal Components Analysis of genotyping data. (a) PCA of the genotyping data from the populations in the HapMap project and GWAS targeting the Turkish population (TUR); (b) PCA of the genotyping data from the GWAS targeting the Turkish population and the individual used for sequencing in this project (IND).

Prior to the sequencing step, we compared the microarray genotyping results of the individual used in this project, who was an anonymous, healthy male claiming to have come from Turkish ancestry for at least three generations, with those obtained from the HapMap project [9] and a recent genome-wide association study targeting Behcet's disease [29]. The latter has utilized 1,215 cases and 1,278 controls from Turkey, genotyped on Illumina's HumanCNV370-Quad v3.0 1 (Illumina®) chip. HapMap populations represent African ancestry in the southwestern USA (ASW); Utah, USA inhabitants with ancestry from northern and western Europe (CEU); Han Chinese in Beijing, China (CHB); Chinese in metropolitan Denver, Colorado, USA (CHD); Gujarati Indians in Houston, Texas, USA (GIH); Japanese in Tokyo, Japan (JPT); Luhya in Webuye, Kenya (LWK); Maasai in Kinyawa, Kenya (MKK); Mexican ancestry in Los Angeles, California, USA (MXL); Tuscans in Italy (TSI); and Yoruba in Ibadan, Nigeria (YRI). We compared the SNP calls coming from the three data sets using Eigenstrat v4.2 [30] to investigate the clustering of individuals based on a principal components analysis (PCA). We then performed PCA analysis only on the Turkish samples used in the Behcet study and the individual used in the current study. In both PCA analyses shown in Figure 1, we utilized only the healthy controls from the Behcet study. Our results show that the individual chosen for WGS represents a typical member of the Turkish population, which differs from the populations used in the HapMap project.

### Trimming, Mapping, and Assembly of the Reads

DNA sequencing generated 1,238,722,496 paired-end reads corresponding to ∼125,111 M bases of data yielding ∼35x coverage. Quality and length-based trimming and filtering dropped 4.44% of the reads eliminating a total 5.03% of total base pairs. The remaining ∼1.18 billion reads (accounting for ∼116,720 M bases) proved to be of high quality (mode of the average read quality Q is ∼38), with sufficient length (mode is ∼100 bp), and included no Ns (Figure S3). Of the high quality reads, 95.28% (∼1.13 billion) were successfully mapped to the reference genome (GRCh37/hg19) covering 99.6% of the bases in the reference genome using the BWA mapping approach. Approximately 50 million unmapped reads (accounting for 4,946 M bases) were assembled using IDBA, which generated 11,654 contigs with lengths ranging between 100 – 43,190 base pairs amounting to ∼10 Mbp of potentially novel sequence. Mean contig length was 856 bp with an N50 of 1,378 bp and an N80 of 497 bp. Of the contigs, 9,486 (∼81%) received a hit in the RefSeq database. Most of the contigs that received a hit were found to be homologues to alternate, reference, or other human sequences (∼97%), while the remaining 313 contigs were found to be homologous to nonhuman primates and other sequences. The 2,168 contigs that were not found to be homologous to any sequences in the RefSeq database represented a total of 927,213 base pairs of assembly with a mean contig length of 427 bp and an N50 of 469 bp. These results are summarized in Table 1.

**Table 1:** Read Sequencing and Analysis Statistics.

**Trimming and Filtering**					
No. of Reads (Raw)	Total Base Pairs (Raw)		No. of Reads (Trimmed and Filtered)		Total Base Pairs (Trimmed and Filtered)
∼1.24×10^9^	∼125×10^9^		∼1.18×10^9^		∼117×10^9^
**Mapping**					
No. of Mapped High Quality Reads	Total Base Pairs Mapped		No. of Unmapped High Quality Reads		Total Base Pairs Unmapped
∼1.13×10^9^	∼112×10^9^		∼50×10^6^		∼5×10^9^
**Assembly of Unmapped Reads**					
No. of Contigs	Total Length of the Assembly (bp)		Min.–Max.–Mean Contig Length (bp)		N50 (bp)
11,654	9,987,256		100–43,190–856		1,378
**Homology Search**					
Contigs Without a Hit	Total Length of Unhit Contigs (bp)		Min.–Max.–Mean Unhit Contig Length		N50 of Unhit Contigs (bp)
2,168 (19%)	927,213		100–9,345–427		469
Contigs With a Hit	Reference Genome	Alternate Assemblies	Other Human Sequences	Non-human primates	Other
9,486 (81%)	983 (8.5%)	7,814 (67.0%)	376 (3.2%)	218 (1.9%)	95 (0.8%)

### SNP Identification

**Figure 2:**
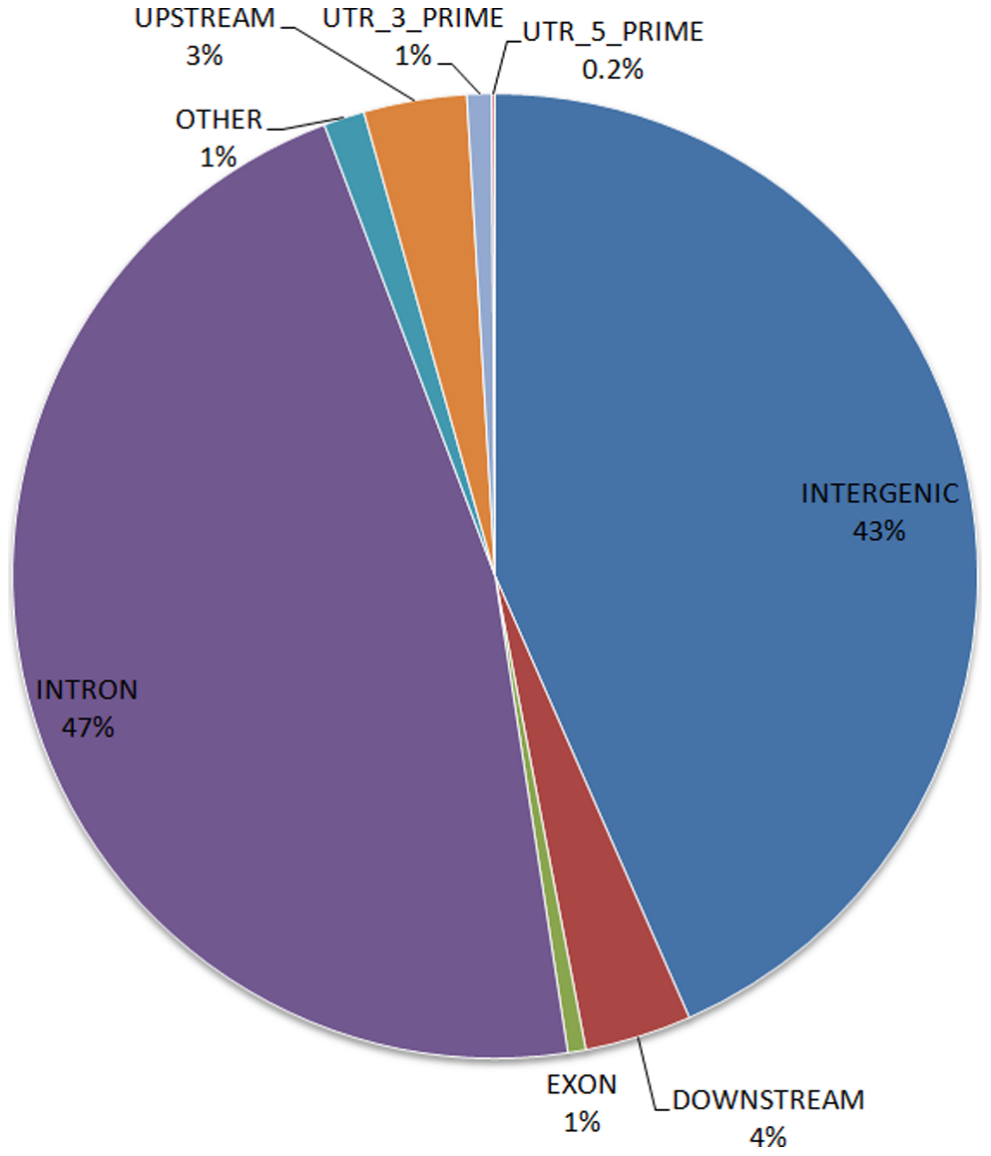
Distribution of the 3,537,794 identified SNPs based on their genomic location.

Casava and GATK workflows identified 3,642,449 and 4,301,769 SNPs, respectively. In order to increase the reliability of our findings, all downstream analysis was performed with SNPs identified by both methods, which resulted in 3,537,794 variants. Of these concordant SNP calls, 97.8% were in agreement with the SNPs called by the genotyping performed on the array, showing high reproducibility. The transitions (2,383,204) transversions (1,154,590), Ts/Tv, ratio was 2.06, and the homozygosity (1,415,123) and heterozygosity (2,122,671) proportions were 40% and 60%, respectively, with both ratios and percentages resembling expected figures in similar studies [14]. Of the SNPs, 47% were in an intronic region; and 43% of the SNPs were in an intergenic region. About 7% of the SNPs were in upstream or downstream regions of a gene and an additional 1.2% of the SNPs were in an untranslated 3′ or 5′ region. Of these SNPs, 29,184 were identified in coding regions with 15,876 synonymous, 13,202 nonsynonymous, and 106 nonsense SNPs. In Figure 2, we show the distribution of the SNPs based on the region in which they were found.

SNPs, where (i) the variant hits a splice acceptor/donor site, (ii) a start codon is changed into a nonstart codon, or (iii) a stop codon is gained or lost due to the variant are categorized as having a “high-impact” effect. There were a total of 259 SNPs with a high-impact effect and 167 of these were found to be on gene sequences. Out of the ∼3.5 million SNPs identified, there were 480,396 potentially novel SNPs that did not exist in dbSNP. Of these potentially novel SNPs, 49% and 41% were in intergenic and intronic regions, respectively. Over 8% were upstream or downstream of a gene, and 2,925 (or 0.5%) of the SNPs were found to be in coding regions. There were 48 nonsense SNPs and a total of 95 SNPs with a high impact. These high-impact SNPs affected 47 genes (45 well characterized). In Table S1, we list these genes along with their effects and annotations. In Table 2, we show the 23 well-characterized genes that were affected by a novel nonsense SNP. Gene annotations were obtained using the GeneALaCart tool of the GeneCards suite (www.genecards.org) [31].

**Table 2:** Annotation for the 23 genes that were affected by a novel nonsense SNP.

Symbol	Descriptions	Chr	Disorder/Disease	Function	Pathway
ABCA9	ATP-binding cassette A9	17	Pseudoxanthoma elasticum	Monocyte differentiation; Lipid homeostasis	ABC transporters
ADCK3	aarF domain containing kinase 3	1	Spinocerebellar ataxia	Protein serine/threonine kinase activity	
ANKRD35	Ankyrin repeat domain-containing protein 35	1		Protein binding	
CAD	CAD trifunctional protein	2	Fibrosarcoma	Aspartate carbamoyltransferase activity	Pyrimidine metabolism; Transcription/Ligand-dependent activation of the ESR1/SP pathway
CDC27	cell division cycle 27	17		Cell cycle checkpoint	Cell cycle_Regulation of G1/S transition
DPRX	Divergent-paired related homeobox	19		Sequence-specific DNA binding TF activity	
FRG2C	FSHD region gene 2 family, member C	3			
GIMAP6	GTPase, IMAP family member 6	7		GTP binding	
HSPBAP1	27 kDa heat shock protein-associated protein 1	3	Intractable epilepsy; Renal carcinoma	Cellular stress response	
HTR2C	5-hydroxytryptamine receptor 1C	X	Schizophrenia; Migraine; Prader-Willi syndrome; Attention deficit hyperactivity disease	Phosphatidylinositol phospholipase C activity	Calcium signaling pathway; Neuroactive ligand-receptor interaction
KBTBD3	BTB and kelch domain-containing protein 3	11		Protein binding	
KRTAP2-2	Keratin-associated protein 2.2	17		Keratin filament	
MLL3	Myeloid/lymphoid leukemia3	7	Leukemia	Methyltransferase activity	Lysine degradation
MYT1	Myelin transcription factor I	20	Dysembryoplastic neuroepithelial tumor; Periventricular leukomalacia	Oligodendrocyte lineage development	
PCNT	Pericentrin	21	Seckel syndrome; Microcephaly	M transition of mitotic cell cycle	Centrosome maturation
PPP2R2B	Protein phosphatase 2, regulatory subunit B	5	Spinocerebellar ataxia	Apoptotic process	mRNA surveillance pathway; Tight junction; Reg'n. of CFTR activity
PROSER1	Proline and serine rich 1	13			
TBCK	TBC1 domain containing kinase	4			
TCP10L2	T-complex 10 like prtn. 2	6	Spina bifida	Cytosol	
TECTA	Tectorin alpha	11	Nonsyndromic deafness; Scotoma; Sensorineural hearing loss	Cell-matrix adhesion	
TFAP2B	Transcription factor AP-2 beta	6	Patent ductus arteriosus; Skeletal muscle neoplasm	Cellular ammonia/urea/creatinine homeostasis	
XIAP	X-linked inhibitor of apoptosis protein	X	Leukemia; Lymphoma	Caspases, apoptosis regulation; inflammation	Ubiquitin mediated proteolysis; SMAC-mediated apoptosis
ZNF778	Zinc finger protein 778	16	KBG syndrome; Learning disability	Zinc ion binding	

### Indel Detection

We identified 713,640 indels, which consisted of 341,382 insertions and 372,258 deletions. Of these indels, 159,593 (or 22%) were found to be novel. The length distribution ranged from -52 bp to 34 bp (see Figure S4 for the histogram of indel lengths) where the average ± standard deviation values were 17.03±9.72 bp for insertions and -26.50±15.15 bp for deletions. Of the indels, 40.8% and 49.5% were in an intergenic and intronic region, respectively. An additional 8.3% were equally divided between upstream and downstream regions of genes; and about 1% were in 3′ and 5′ untranslated regions, the majority (∼95%) being in the 3′ UTR. Only 50 and 53 indels were in a splice site acceptor and donor regions, respectively; and 1,934 (or 0.2%) affected a coding region. Of these, 246 indels caused a frame shift, while 104 resulted in a codon deletion and 75 resulted in a codon insertion. When we imposed a window-based filtering such that no two indels co-occurred within 20 bp of each other, we identified 655,195 indels, out of which 123,478 (or 19%) were novel.

We performed Sanger sequencing on 20 regions containing 20 predicted indels and validated 18 of these indels. We randomly selected the indels for validation with the constraints that they overlapped with coding sequences in possibly detrimental ways and showed different homo/heterozygosity status in a ratio of ∼2∶1, as observed in previous studies [4]. Seventeen of the 20 indels used for validation were overlapping with known genes, and 3 were overlapping with predicted gene regions. Fourteen indels were homozygous, and 6 were heterozygous. The 20 indels represented 3 frame-shift deletions, 5 frame-shift insertions, 3 nonframe-shift deletions, 8 nonframe-shift insertions and 1 stop-gain SNV all overlapping with coding sequencing in potentially detrimental ways. We list the details of the 20 indels used for validation and utilized forward and reverse primers in Table S2 and Table S3.

### Structural Variant Discovery

Employed read-depth and paired-end CNV/SV discovery methods [26], [27] identified 9,109 such events including 7302 deletions, 1663 duplications, and 144 insertions. Length normalized distribution of these calls followed a uniform distribution across chromosomes. On average, we observed 3.11 CNV/SV events per chromosome per million base pairs with a standard deviation of 0.77 (Figure S5). Of the predicted CNV/SV calls, 58.5% overlapped with the structural variants identified as part of the 1000 Genomes Project. The length distribution of the total and novel CNV/SV events revealed that 3,820 out of 9,109 total (or 42%) and 1,786 out of 3,780 novel (or 48%) events were less than 1 Kbp (Figure S6). When we compared the CNV/SV events called by two different algorithms, we identified 1,629 concordant, high-confidence calls. Of these high confidence calls, 1,223 (or 75%) overlapped with CNV/SVs identified as part of the 1000 Genomes Project. We also verified the CNV/SV calls with the results of the SNP chip data and found 394 concordant calls.

## Discussion

In this paper, we present high-depth coverage (∼35×) and detailed analysis of the whole genome sequence of a Turkish individual. Although whole genome human sequencing is almost routinely done, very few of these efforts provide high coverage and analysis; and various populations are not included in large consortium efforts. Therefore, we believe the current study provides a reference data set in understanding human genome variation on a large scale and a population-dependent context and is an initial step in exploring Turkish genomic features. Despite its importance as the first Turkish whole genome analysis, we acknowledge that further studies are required to validate and generalize our findings to the population scale. Nevertheless, we believe the data presented here will provide a cornerstone for such studies and enrich the analysis of human genomic variation across diverse populations.

Our genotyping results from chip and sequence data, and the comparative analysis of these with HapMap and other chip genotyping results suggest that the sequenced individual represents a typical member of the Turkish population. The sequencing analysis proves that the data generated is of high quality as only 5% of the total sequence is filtered out, and the remaining reads almost completely cover the reference human genome. High N50 values indicate successful assembly of unmapped reads, which also resulted in ∼1 Mbp of unmapped human genome sequence that did not reveal any homology in the RefSeq database. SNP identification rendered high reproducibility with ∼98% consistency between the sequencing and microarray genotyping results and validated about 86% of identified SNPs in the dbSNP database. We believe the remaining 480,396 potentially novel SNPs contribute to understanding the human genomic variation and the relationship between genotypes and phenotypes.

The length distribution of the 9,109 identified CNV/SV events showed peaks at the 300 bp and 6,000 bp marks potentially representing short and long interspersed elements, respectively. A similar trend is seen in the 1,629 high confidence calls (Figure S6). The relatively high number of CNV/SV calls are due to the inclusion of short variants (variants smaller than 1 Kbp), which constitute 42% of total calls and 48% of novel calls. We validated CNV/SV events that fall within a size range which can be validated with the SNP chip. We have been able to verify 394 CNV/SV calls where the majority of the variants were greater than 10 kbp in length. We found 3,780 CNV/SV calls that were not identified in the 1000 Genomes Project and may be variants potentially specific to the Turkish population.

In order to position our results in a better population genetics context, we compared the SNPs identified in the sequenced Turkish individual to the SNPs found in Utah, USA inhabitants with ancestry from Europe (CEU) and to the SNPs found in Han Chinese in Beijing, China (CHB). Both CEU and CHB populations were included in the HapMap project, which constitute the source of the identified SNPs in these populations used in our analysis. From a historical perspective, precursors of the Turks originated in Central Asia and Turks are known to have been inhabitants of regions that are parts of modern day China. Turks' migration toward the West ended up mostly in Anatolia with minor settlements in Europe. Turks' presence in Europe was later expanded throughout the Ottoman era. We, therefore, performed our comparative analysis with CEU and CHB, which are the two most closely related populations to the Turkish population with available large-scale genetic data. We found 665,032 SNPs commonly shared by the three populations, which corresponds to 47% of all of the CEU SNPs and 50% of all the CHB SNPs (Figure S7). SNPs exclusively shared by the Turkish and CEU populations were 3% of the total CEU SNPs (43,592 out of 1,412,090 SNPs) while SNPs exclusively shared by the Turkish and CHB populations were 1% of the total CHB SNPs (15,657 out of 1,328,223 SNPs). Although more evidence is needed to make conclusive remarks, our results may suggest that the Turkish population is almost equidistant to the CEU and CHB populations, being slightly closer to the CEU.

Novel SNPs predicted by our results have the potential to explain genetic features specific to the Turkish population. The 23 well-characterized genes that were affected by the novel nonsense SNPs identified in this study were found to affect 25 different phenotypes listed in OMIM, potentially leading to additional genetic disease mechanisms (see Table 2). We used Ingenuity Software Knowledge Base (IKB), (Redwood City, CA) to further identify networks that explain underlying interactions for the 47 genes that were affected by a high-impact novel SNP. The biological functions identified by IKB are grouped in 66 categories. Out of these categories, two of the most significant ones were hereditary disorders and neurological diseases. The former included 14 disorders, 3 of which were X-linked via the gene XIAP; and the latter involved 27 diseases, most notably due to HTR2C. These results are summarized in Table S4.

**Figure 3:**
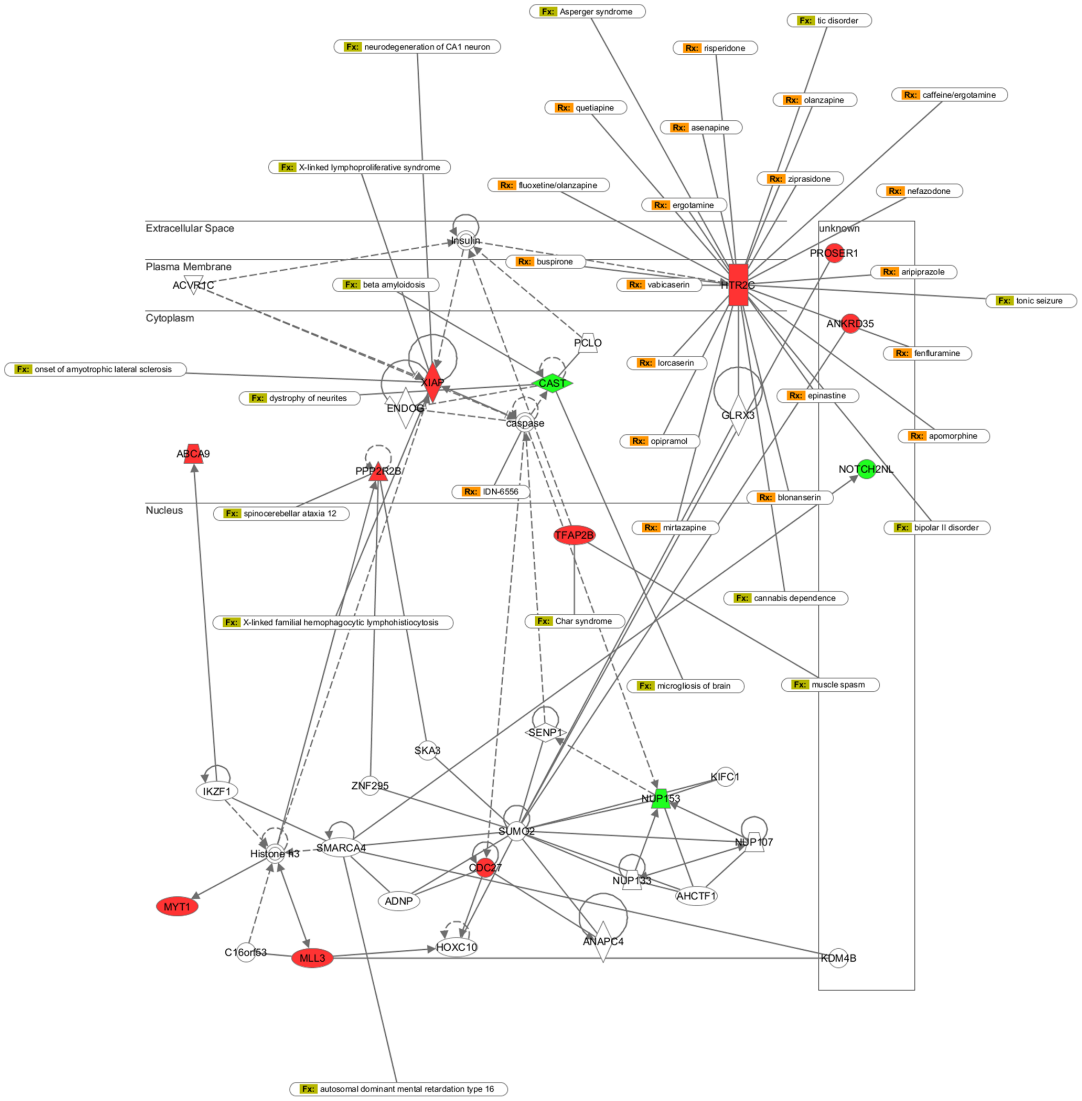
Ingenuity Network analysis of 45 genes affected by a high impact novel SNP. Genes indicated by red are affected by a nonsense SNP and genes indicated by green are affected by an SNP targeting a splice site donor/acceptor region. Drug targets, hereditary and neurological disorders/diseases are indicated where applicable.

IKB analysis revealed three interaction networks that included 15, 13, and 3 of the 47 genes. In Figure 3 and Figure S8, we show the first two networks where corresponding drug and hereditary and neurological disorders/diseases are overlaid on the networks. In this representation, we show the subcellular layout; and for the network shown in Figure 3, HTR2C and XIAP seem to play central roles at the membrane and cytoplasm levels. We observe a downstream effect of HTR2C on SMT3 suppressor of mif two 3 homolog 2 (SUMO2) through glutaredoxin 3 (GLRX3). It has been shown that 5-hydroxytryptamine receptors are associated with protein networks involved in synaptic localization along with multidomain proteins such as GLRX3 [32]. GLRX3 may participate in the inhibition of apoptosis and play a role in cellular growth, regulating the function of the thioredoxin system; and contains subunits that may serve as a redox sensor [33]. SUMO2, which binds with GLRX3 [34], is known to be involved in a number of processes like apoptosis, protein stability, and transcriptional regulation. On the other hand, SUMO2 is active in acetylation of histone H3 [35] through SMARCA4 [36]; and XIAP is involved in methylation of histone H3 [37]. This cascade of events may suggest a mechanism in which two central genes affected by novel nonsense SNPs identified in this study, XIAP and HTR2C, are involved in various neurological diseases such as amyotrophic lateral sclerosis [38]. In the other interaction network identified by IKB, we see a key role played by ubiquitin C (UBC), which is linked to the genes affected by novel high impact SNPs. The suggested outcomes involve various neurological disorders in addition to tumorigenesis and blastomas (Figure S8). Overall, we believe that functional and systems level analysis of population-dependent genomic variations may shed light on disease mechanisms as demonstrated here.

### Data Accession

The whole genome sequencing reads have been deposited in the NCBI Short Read Archive (SRA) database under the accession number SRA056442. SNPs and indels have been deposited in the NCBI dbSNP database with the handle ID “BILGI_BIOE”. CNV/SV calls have been deposited in the NCBI dbVar database with project ID PRJNA171612.

## Supporting Information

Figure S1 **QC gel image (0.8% agarose) of gDNA sample (1) compared to molecular weight marker (M).** Sample quality was considered to be acceptable if the gDNA supplied a single visible band while lacking any significant degradation products (degraded DNA seen as smear of small fragments).
(TIF)

Figure S2 **Library quality.** The electropherogram for the generated library displaying expected yield and size.
(TIF)

Figure S3 **Quality statistics for the forward reads only (almost identical results are obtained for the reverse reads) following trimming and filtering.** a) Average base quality with respect to the position of the base in the read; b) Histogram of the sequence lengths; c) histogram of the average quality scores of the reads; d) Ns seen in the reads with respect to the position of the base in the read.
(TIF)

Figure S4 **Length distribution of the identified 713, 640 indels.**
(TIF)

Figure S5 **Distribution of 9,109 identified CNV/SV calls across chromosomes.** A) Number of CNV/SV events; B) Length normalized (per million base pairs) CNV/SV events.
(TIF)

Figure S6 **Length distribution of 9,109 identified, 3870 novel, and 1629 high confidence CNV/SV calls.** Note that the bin size for SVs less than 10 Kbp is 200 bp while the bin size for SVs more than 10 Kbp is 2 Kbp. The last bar in the graphs on the right-hand column represents SVs more than 250 Kbp.
(TIF)

Figure S7 **Overlap of SNPs identified in the Turkish individual used in the manuscript (TUR); Utah, USA inhabitants with ancestry from Europe (CEU); and Han Chinese in Beijing, China (CHB).**
(TIF)

Figure S8 **IKB Network analysis of 45 genes affected by a high impact novel SNP.** Genes indicated by red are affected by a nonsense SNP and genes indicated by green are affected by a SNP targeting a splice site donor/acceptor region. Hereditary and Neurological Disorders/Diseases are indicated where applicable.
(TIF)

Table S1 **45 well characterized genes that were affected by a high-impact SNP.** Effect Types: 1: Stop gained; 2: Splice site acceptor; 3: Splice site donor; 4: Stop lost; 5: Start lost.
(PDF)

Table S2 **20 predicted indels used for validation by Sanger Sequencing (V: Validated NV: Not Validated).**
(PDF)

Table S3 **Forward and reverse primers used in Sanger Sequencing.**
(PDF)

Table S4 **Biological Function categories known to involve 45 well characterized genes that were affected by a high-impact SNP.**
(PDF)
