# Supplementary figures and images for: Whole Genome Sequence of a Turkish Individual
Source: PLoS One. 2014 Jan 9;9(1):e85233. doi: 10.1371/journal.pone.0085233 (PMC3887021; doi:10.1371/journal.pone.0085233)

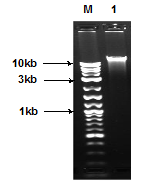

Supplement: Figure S1 — QC gel image (0.8% agarose) of gDNA sample (1) compared to molecular weight marker (M). Sample quality was considered to be acceptable if the gDNA supplied a single visible band while lacking any significant degradation products (degraded DNA seen as smear of small fragments). (TIF) [file pone.0085233.s001.tif]

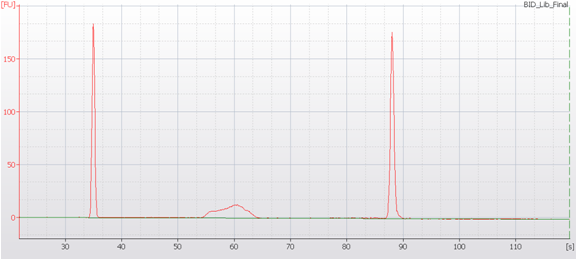

Supplement: Figure S2 — Library quality. The electropherogram for the generated library displaying expected yield and size. (TIF) [file pone.0085233.s002.tif]

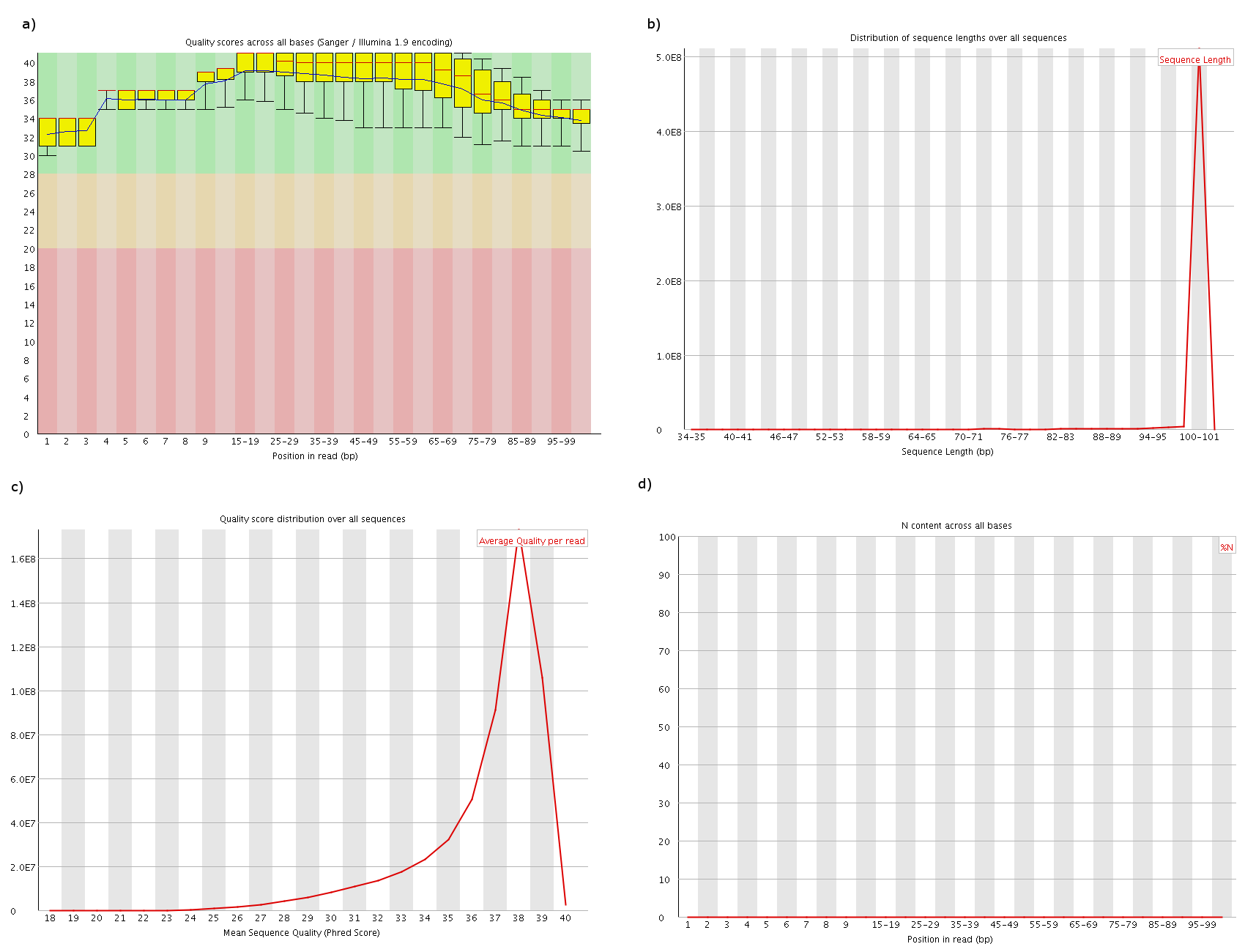

Supplement: Figure S3 — Quality statistics for the forward reads only (almost identical results are obtained for the reverse reads) following trimming and filtering. a) Average base quality with respect to the position of the base in the read; b) Histogram of the sequence lengths; c) histogram of the average quality scores of the reads; d) Ns seen in the reads with respect to the position of the base in the read. (TIF) [file pone.0085233.s003.tif]

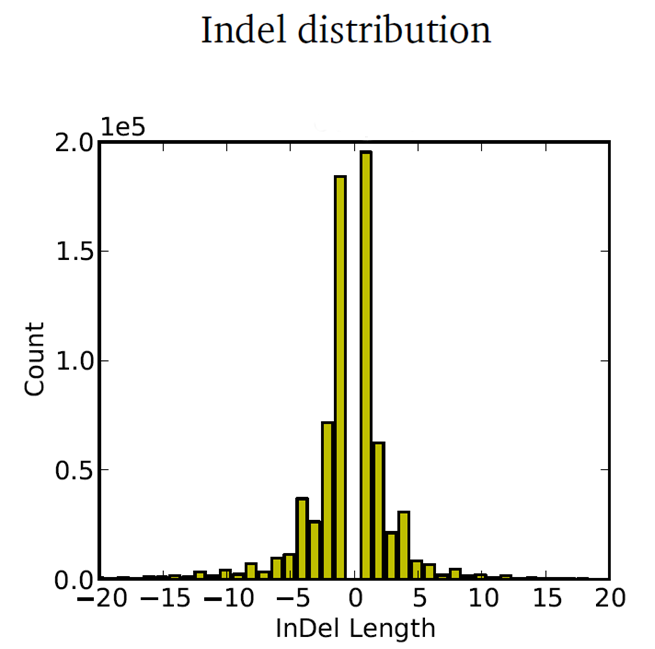

Supplement: Figure S4 — Length distribution of the identified 713, 640 indels. (TIF) [file pone.0085233.s004.tif]

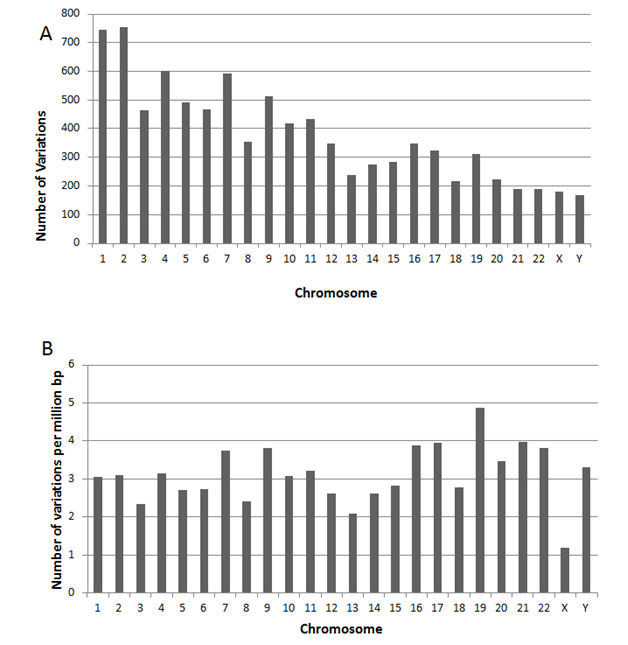

Supplement: Figure S5 — Distribution of 9,109 identified CNV/SV calls across chromosomes. A) Number of CNV/SV events; B) Length normalized (per million base pairs) CNV/SV events. (TIF) [file pone.0085233.s005.tif]

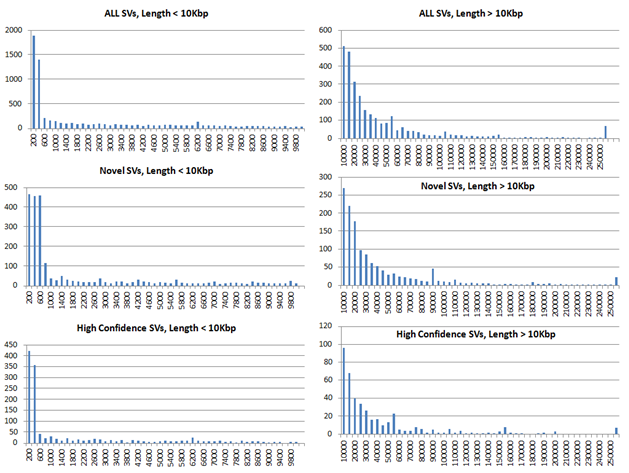

Supplement: Figure S6 — Length distribution of 9,109 identified, 3870 novel, and 1629 high confidence CNV/SV calls. Note that the bin size for SVs less than 10 Kbp is 200 bp while the bin size for SVs more than 10 Kbp is 2 Kbp. The last bar in the graphs on the right-hand column represents SVs more than 250 Kbp. (TIF) [file pone.0085233.s006.tif]

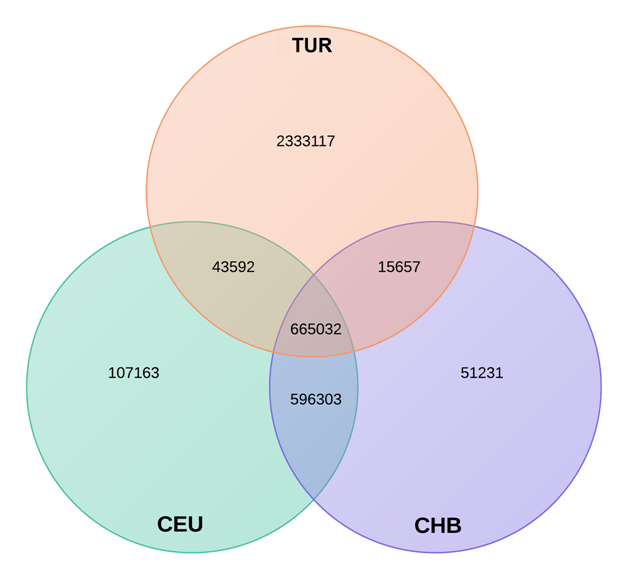

Supplement: Figure S7 — Overlap of SNPs identified in the Turkish individual used in the manuscript (TUR); Utah, USA inhabitants with ancestry from Europe (CEU); and Han Chinese in Beijing, China (CHB). (TIF) [file pone.0085233.s007.tif]

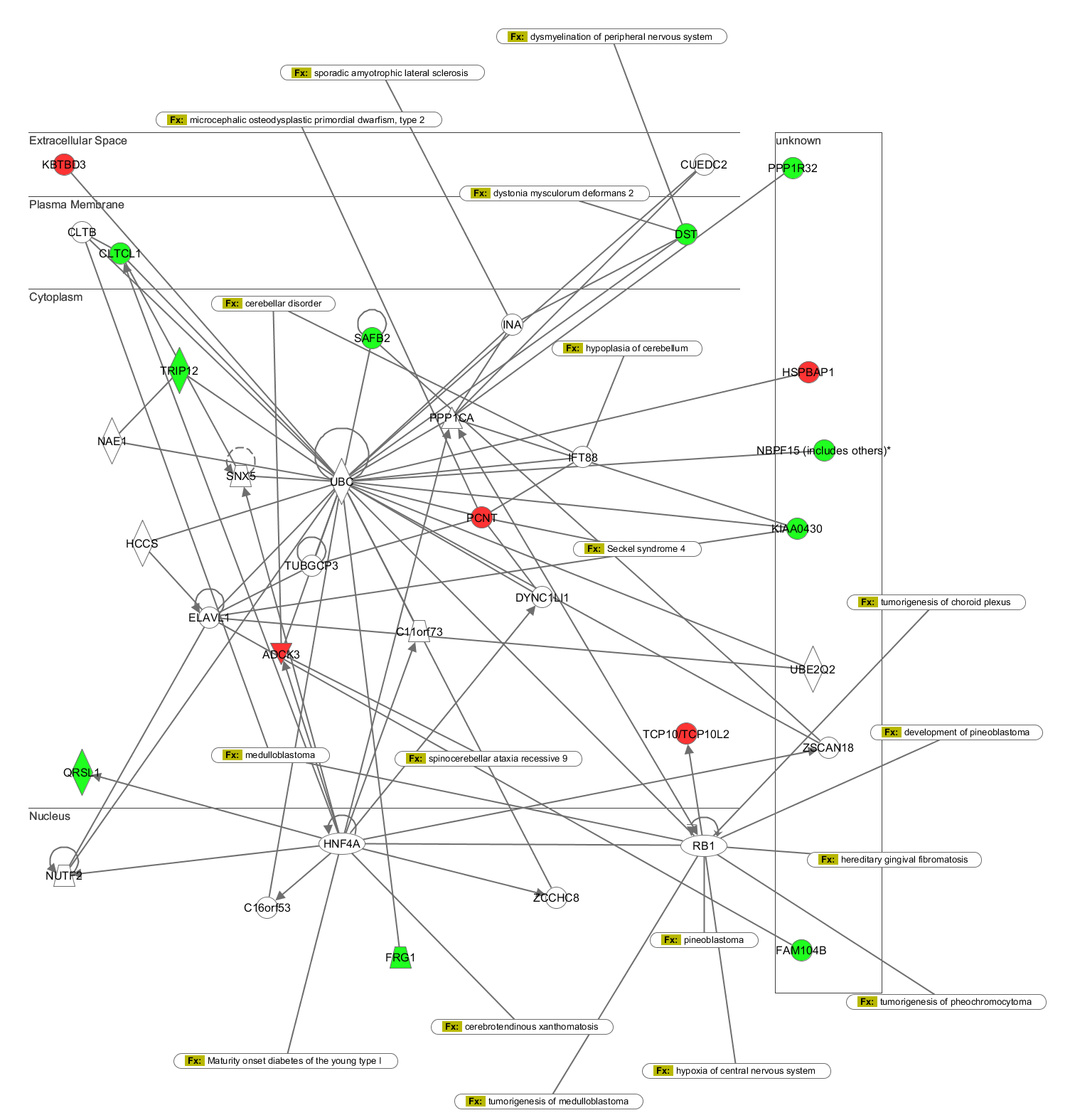

Supplement: Figure S8 — IKB Network analysis of 45 genes affected by a high impact novel SNP. Genes indicated by red are affected by a nonsense SNP and genes indicated by green are affected by a SNP targeting a splice site donor/acceptor region. Hereditary and Neurological Disorders/Diseases are indicated where applicable. (TIF) [file pone.0085233.s008.tif]
