# Supplementary material for: Whole Genome Sequence of a Turkish Individual
Source: PLoS One. 2014 Jan 9;9(1):e85233. doi: 10.1371/journal.pone.0085233 (PMC3887021; doi:10.1371/journal.pone.0085233)
Supplement: Table S1 — 45 well characterized genes that were affected by a high-impact SNP. Effect Types: 1: Stop gained; 2: Splice site acceptor; 3: Splice site donor; 4: Stop lost; 5: Start lost. (PDF) [file pone.0085233.s009.pdf]

Table S1. 45 well characterized genes that were affected by a high-impact SNP. Effect Types: 1: Stop gained; 2: Splice site acceptor; 3: Splice site donor; 4: Stop lost; 5: Start lost.

Symbol	Descriptions	Chr	Disorder/Disease	Function	Pathway	Effect
ABCA9	ATP-binding cassette A9	17	Pseudoxanthoma elasticum	Monocyte differentiation; Lipid homeostasis	ABC transporters	1
ADCK3	aarF domain containing kinase 3	1	Spinocerebellar ataxia	Protein serine/threonine kinase activity		1
ANKRD35	Ankyrin repeat containing protein 35	1		Protein binding		1
AQP7P3	Aquaporin 7 pseudogene 3	9		Water transport		2
CAD	CAD trifunctional protein	2	Fibrosarcoma	Aspartate carbamoyltransferase activity	Pyrimdne metabolism; Transcription/Ligand-dependent activation of ESR1/SP pathway	1
CAST	Calpain inhibitor	5	Vascular disease; Alzheimer's disease	Endopeptidase inhibitor activity		2
CDC27	cell division cycle 27	17		Cell cycle checkpoint	Cell cycle_Regulation of G1/S transition	1
CES1	Carboxylesterase 1	16	CES 1 deficiency	carboxylesterase activity	Drug metabolism; Cholesterol and Sphingolipids transport	3
CLTCL1	Clathrin, heavy chain-like 1	22	Tetralogy of Fallot; Ventricular septal defect; trioventricular septal defect; Pulmonary valve stenosis	Receptor-mediated endocytosis	Lysosome; Endocytosis; Endocrine-regulated calcium reabsorption; Huntington's disease; Bacterial invasion of epithelial cells	3
DEFB108B	Beta-defensin 8	11		defense response to bacterium		5
DPRX	Divergent-paired related homeobox	19		Sequence-specific DNA binding transcription factor activity		1
DST	Dystonia musculorum protein	6	Bullous pemphigoid; Epidermolysis bullosa	Microtubule cytoskeleton organization	Cytoskeleton remodeling, Keratin/Neuro filaments	2
FAM104B	Family with sequence similarity 104, member B	X				3
FRG1	FSHD region gene-1	4	Facioscapulohumeral muscular dystrophy; Suppurative lymphadenitis	Nuclear mRNA splicing via spliceosome		3
FRG1B	FSHD region gene 1 family, member B	20				2
FRG2C	FSHD region gene 2 family, member C	3				1
GIMAP6	GTPase, IMAP family member 6	7		GTP binding		1
HEATR7B2	HEAT repeat family member 7B2	5		Binding		2
HSPBAP1	27 kDa heat shock protein-associated protein 1	3	Intractable epilepsy; Renal carcinoma	Cellular stress response		1
HTR2C	5-hydroxytryptamine receptor 1C	X	Schizophrenia; Migraine; Prader-Willi syndrome; Attention deficit hyperactivity	Phosphatidylinositol phospholipase C activity	Calcium signaling pathway; Neuroactive ligand-receptor interaction	1

KBTBD3	BTB and kelch domain-containing protein 3	11		Protein binding		1
KIAA0430	Limkain-b1	16		Nucleotide binding		2
KRTAP2-2	Keratin-associated protein 2.2	17		Keratin filament		1
MLL3	Myeloid/lymphoid leukemia 3	7	Leukemia	Methyltransferase activity	Lysine degradation	1
MST1P2	Macrophage stimulating 1 pseudogene 2	1				2
MYT1	Myelin transcription factor I	20	Dysembryoplastic neuroepithelial tumor; Periventricular leukomalacia	Oligodendrocyte lineage development		1
NBPF1	Neuroblastoma breakpoint family member 1	1	Shipyard eye; Gastroenteritis	Cytoplasm		4
NBPF15	Neuroblastoma breakpoint family, member 15	1		Cytoplasm		2
NOTCH2NL	Notch 2 N-terminal like	1	Alagille syndrome; Hajdu-Cheney syndrome; Glioma	Calcium ion binding	Notch signaling pathway	3
NUP153	Nuclear pore complex protein	6	Infectious mononucleosis; Hemophagocytic lymphohistiocytosis	DNA binding	Host Interactions of HIV factors	3
OR8U8	Olfactory receptor 8U8	11		G-protein coupled olfactory receptor activity	Olfactory transduction	3
PCNT	Pericentrin	21	Seckel syndrome; Microcephaly	M transition of mitotic cell cycle	Centrosome maturation	1
PPP1R32	Protein phosphatase 1 regulatory subunit	11		Phosphatase binding		2
PPP2R2B	Protein phosphatase 2, regulatory subunit B	5	Spinocerebellar ataxia	Apoptotic process	mRNA surveillance pathway; Tight junction; Regulation of CFTR activity	1
PROSER1	Proline and serine rich 1	13				1
QRSL1	Glutamyl-amidotransferase subunit A	6	Lymphogranuloma venereum; Intestinal volvulus; Trachoma	Carbon-nitrogen ligase activity		2
SAFB2	Scaffold attachment factor B2	19	Breast Cancer	Nucleotide binding		2
TBCK	TBC1 domain containing kinase	4				1
TCP10L2	T-complex 10-like protein 2	6	Spina bifida	Cytosol		1
TECTA	Tectorin alpha	11	Nonsyndromic deafness; Scotoma; Sensorineural hearing loss	Cell-matrix adhesion		1
TFAP2B	Transcription factor AP-2 beta	6	Patent ductus arteriosus; Skeletal muscle neoplasm	Cellular ammonia/urea/creatinine homeostasis		1
TRIP12	Thyroid receptor-interacting protein 12	2	Chronic dacryocystitis; Heart disease	Ubiquitin-protein ligase activity	Ubiquitin mediated proteolysis	3
WASH2P	Protein FAM39B	2	Fission of tubules			3
XIAP	X-linked inhibitor of apoptosis protein	X	Leukemia; Lymphoma	Caspases, apoptosis regulation; inflammatory signaling	Ubiquitin mediated proteolysis; SMAC-mediated apoptotic r.	1
ZNF778	Zinc finger protein 778	16	KBG syndrome; Learning disability	Zinc ion binding		1
