# Supplementary material for: Whole Genome Sequence of a Turkish Individual
Source: PLoS One. 2014 Jan 9;9(1):e85233. doi: 10.1371/journal.pone.0085233 (PMC3887021; doi:10.1371/journal.pone.0085233)
Supplement: Table S2 — 20 predicted indels used for validation by Sanger Sequencing (V: Validated NV: Not Validated). (PDF) [file pone.0085233.s010.pdf]

Table S2: 20 predicted indels used for validation by Sanger Sequencing (**V**: Validated **NV**: Not Validated)

Variant	Gene Name	Accession	Chr	Start	End	Reference	Observation	Heterozygosity Status	Sanger Seq.
Frameshift Deletion	ZNF717	NM_001128223	3	75790773	75790783	GGCTGCTGTAG	-	Het	V
Frameshift Deletion	OR4L1	NM_001004717	14	20528449	20528467	CATAGATTTGCTCACTGAC	-	Het	V
Frameshift Deletion	DCHS2	NM_001142552	4	155244402	155244405	TTTG	-	Hom	V
Frameshift Insertion	SETBP1	NM_001130110	18	42456670	42456670	-	TCTT	Hom	V
Frameshift Insertion	ZNF717	NM_001128223	3	75786555	75786555	-	TG	Het	V
Frameshift Insertion	C11orf40	NM_144663	11	4592708	4592708	-	AG	Hom	NV
Frameshift Insertion	ATRNL1	NM_001276282	10	116931101	116931101	-	TT	Hom	V
Frameshift Insertion	C11orf40	NM_144663	11	4592706	4592706	-	AC	Hom	V
Nonframeshift Deletion	ZNF717	NM_001128223	3	75786315	75786323	CTACATTCT	-	Het	V
Nonframeshift Deletion	PRUNE2	NM_015225	9	79318376	79318390	GTGACAGCCTGCAAC	-	Hom	V
Nonframeshift Deletion	KLRF1	NM_016523	12	9994446	9994448	TGT	-	Hom	V
Nonframeshift Insertion	MUC4	NM_018406	3	195518112	195518112	-	GTCTCCTGCGTAACA	Hom	V
Nonframeshift Insertion	C8orf59	NM_001099671	8	86126827	86126827	-	AACATT	Hom	V
Nonframeshift Insertion	SYNJ1	NM_001160306	21	34003928	34003928	-	AGTATT	Hom	V
Nonframeshift Insertion	USP40	NM_018218	2	234429739	234429739	-	TTC	Hom	NV
Nonframeshift Insertion	OR5P2	NM_153444	11	7818383	7818383	-	ATATGGTTACCAGGTAGATGC	Het	V
Nonframeshift Insertion	ZNF714	NM_182515	19	21299774	21299774	-	ATA	Hom	V
Nonframeshift Insertion	C18orf25	NM_001008239	18	43833701	43833701	-	CTG	Hom	V
Nonframeshift Insertion	CCDC180	NM_020893	9	100092968	100092968	-	GAGGAG	Hom	V
Stopgain SNV	RETNLB	NM_032579	3	108475993	108475993	-	GGGGATTA	Het	V
