# Supplementary material for: Whole Genome Sequence of a Turkish Individual
Source: PLoS One. 2014 Jan 9;9(1):e85233. doi: 10.1371/journal.pone.0085233 (PMC3887021; doi:10.1371/journal.pone.0085233)
Supplement: Table S3 — Forward and reverse primers used in Sanger Sequencing. (PDF) [file pone.0085233.s011.pdf]

Table S3: Forward and reverse primers used in Sanger Sequencing

Sequence (5'->3')			Template strand	Length	Start	Stop	Tm	GC%	Self comp.	Self 3' compl.
**chr3:195517812 -195518412**	Forward primer	GCTGAGGTCACTGGGGTAGA	Plus	20	137	156	60.61	60.00	4.00	0.00
	Reverse primer	TCCAGGAACCACAGAGGACA	Minus	20	562	543	60.11	55.00	5.00	0.00
	Product length	426								
**chr3:75786015-75786623**	Forward primer	TGTAGGTTGTGTGTTCAAGGGA	Plus	22	1	22	59.49	45.45	3.00	0.00
	Reverse primer	CCTCAGTACGCATCAGGGGA	Minus	20	444	425	61.04	60.00	4.00	0.00
	Product length	444								
**chr8:86126527-86127127**	Forward primer	TGTAAAGTGAAGGATGTAAACGAGG	Plus	25	5	29	59.07	40.00	2.00	0.00
	Reverse primer	TCAAAGCCTGTAAGTTGAGTGT	Minus	22	554	533	57.85	40.91	4.00	0.00
	Product length	550								
**chr3:75790473-75791083**	Forward primer	GCCCTGGTAATGAGAAACAATGGA	Plus	24	45	68	61.11	45.83	3.00	0.00
	Reverse primer	GGCTGATGCTTTGACTTGAGGA	Minus	22	583	562	60.88	50.00	2.00	0.00
	Product length	539								
**chr21:34003628 -34004228**	Forward primer	CACTGAAAGGATTTGTCCTGGT	Plus	22	1	22	58.51	45.45	4.00	1.00
	Reverse primer	TTTACATGCTTGCCCCTAAAAG	Minus	22	411	390	57.20	40.91	4.00	1.00
	Product length	411								
**chr2:234429439 -234430039**	Forward primer	AGCTGACTTCCTAAGAGCTGAA	Plus	22	75	96	58.83	45.45	4.00	0.00
	Reverse primer	ACTGCCTAACATTACCCTGCAAA	Minus	23	482	460	60.50	43.48	4.00	0.00
	Product length	408								

**chr11:7818083-7818683**	Forward primer	TTGCCACAAAGCGGTCATAG	Plus	20	44	63	58.84	50.00	3.00	0.00
	Reverse primer	TCATTAGGGTTCATTTAGTCAGCA	Minus	24	444	421	57.94	37.50	2.00	0.00
	Product length	401								
**chr18:42456370-42456970**	Forward primer	TCTAAGTCAGCCTCCTCATTTTCT	Plus	24	135	158	59.22	41.67	3.00	0.00
	Reverse primer	CTCTTCAGGGCAGCATTGTGA	Minus	21	548	528	60.61	52.38	3.00	1.00
	Product length	414								
**chr3:75786255-75786855**	Forward primer	TGAGTGAGTCCCCTGATGCG	Plus	20	177	196	61.61	60.00	3.00	2.00
	Reverse primer	TCGCAAGTCATTCCTCACCA	Minus	20	549	530	59.32	50.00	2.00	0.00
	Product length	373								
**chr11:4592408-4593008**	Forward primer	TGCCACAGCACAGTAACACC	Plus	20	161	180	60.82	55.00	3.00	0.00
	Reverse primer	GCCATTGACCAGCAGTAAAGCA	Minus	22	594	573	61.72	50.00	3.00	0.00
	Product length	434								
**chr10:116930801-116931401**	Forward primer	TGGAGTTGGATAGTAGAGATGTTGT	Plus	25	158	182	59.04	40.00	3.00	0.00
	Reverse primer	GCATAGTAAAGAGACTGGGATAGTT	Minus	25	592	568	57.94	40.00	3.00	0.00
	Product length	435								
**chr19:21299474-21300074**	Forward primer	TGGCATCTTGCTTATGTAGTTTGT	Plus	24	38	61	58.99	37.50	3.00	0.00
	Reverse primer	ACTCTCTTGTGTCTAGTAAGGGT	Minus	23	575	553	58.00	43.48	4.00	0.00
	Product length	538								
**chr18:43833401-43834001**	Forward primer	ACTTTTATGTACTGCCATGTTGTT	Plus	24	29	52	57.35	33.33	4.00	0.00
	Reverse primer	ACCAAGAACTCCCATCATTCATTC	Minus	24	513	490	59.05	41.67	3.00	0.00
	Product length	485								

**chr11:4592406-4593006**	Forward primer	TGCCACAGCACAGTAACACC	Plus	20	163	182	60.82	55.00	3.00	0.00
	Reverse primer	GCCATTGACCAGCAGTAAAGCA	Minus	22	596	575	61.72	50.00	3.00	0.00
	Product length	434								
**chr9:79318076-79318690**	Forward primer	TGCTTTTACTGATGGTGTGCCT	Plus	22	30	51	60.49	45.45	2.00	0.00
	Reverse primer	TCTCTGAACTCGGCTTGGGT	Minus	20	466	447	60.83	55.00	3.00	0.00
	Product length	437								
**chr14:20528149-20528767**	Forward primer	CTGGAACCTTTCTGAGTTGAGT	Plus	22	32	53	57.92	45.45	3.00	1.00
	Reverse primer	ATCGCAAACCCCTTTAGCAG	Minus	20	492	473	58.54	50.00	3.00	1.00
	Product length	461								
**chr12:9994146-9994748**	Forward primer	TCCCAAGGACCATTATTTGTGT	Plus	22	88	109	57.60	40.91	4.00	0.00
	Reverse primer	ACTGCAAAGATGCCATAACCCT	Minus	22	444	423	60.29	45.45	5.00	0.00
	Product length	357								
**chr9:100092668-100093268**	Forward primer	GACATGACCAGAAGTGAGGAA	Plus	21	2	22	57.31	47.62	4.00	0.00
	Reverse primer	CACTGAAGACCCCAGCTTACA	Minus	21	473	453	59.65	52.38	4.00	0.00
	Product length	472								
**chr4:155244102-155244705**	Forward primer	TCCTGCCACCCATCTCTCTA	Plus	20	110	129	59.36	55.00	3.00	2.00
	Reverse primer	CCAGGCTGCAGTGAGCTATC	Minus	20	466	447	60.53	60.00	6.00	2.00
	Product length	357								
**chr3:108475693-108476293**	Forward primer	TACCATTCTCCTCCACCTCTTC	Plus	22	7	28	58.62	50.00	2.00	0.00
	Reverse primer	TACTGATGACGCTGTTCTTCCA	Minus	22	481	460	59.44	45.45	3.00	0.00
	Product length	475								
