# Supplementary material for: Whole Genome Sequence of a Turkish Individual
Source: PLoS One. 2014 Jan 9;9(1):e85233. doi: 10.1371/journal.pone.0085233 (PMC3887021; doi:10.1371/journal.pone.0085233)
Supplement: Table S4 — Biological Function categories known to involve 45 well characterized genes that were affected by a high-impact SNP. (PDF) [file pone.0085233.s012.pdf]

**Table S4**: Biological Function categories known to involve 45 well characterized genes that were affected by a high-impact SNP.

| Category | Functions Annotation | Molecules |
|---|---|---|
| **Amino Acid Metabolism** | secretion of GABA | HTR2C |
| **Auditory and Vestibular System Development and Function** | sensitivity of basilar membrane | TECTA |
| | abnormal morphology of otolithic membrane | TECTA |
| | quantity of otoliths | TECTA |
| | abnormal morphology of enlarged otoliths | TECTA |
| | abnormal morphology of tectorial membrane | TECTA |
| **Auditory Disease** | childhood onset deafness DFNB8 | TECTA |
| **Behavior** | Stereotypy | HTR2C |
| **Cancer** | survival of pancreatic cancer cells | XIAP |
| | survival of carcinoma cells | XIAP |
| | growth of lung tumor | XIAP |
| | malignant cutaneous melanoma cancer | CDC27,MLL3, TECTA |
| | childhood acute lymphoblastic leukemia | XIAP |
| | tumorigenesis of fibroblast cell lines | CAST |
| | Cancer | CAD,CDC27, CES1,CLTCL1,DST,MLL3,PPP2R2B,SAFB2,TECTA,TFAP2B,XIAP |
| | alveolar rhabdomyosarcoma | TFAP2B |
| **Carbohydrate Metabolism** | hydrolysis of inositol phosphate | HTR2C |
| | regulation of D-glucose | HTR2C |
| **Cardiovascular Disease** | congestive heart failure | TFAP2B,XIAP |
| | vascular dementia | HTR2C |
| | Hemopericardium | XIAP |
| **Cell Cycle** | mitotic exit of cervical cancer cell lines | MYT1 |
| | re-entry into cell cycle progression of fibroblast cell lines | CAST |
| | metaphase/anaphase transition | CDC27 |
| | mitochondrial fission | PPP2R2B |

| | | |
|---|---|---|
| | cell cycle progression | CAST,CDC27,CLTCL1,MYT1,PPP2R2B |
| | cell cycle progression of colon cancer cell lines | CDC27 |
| **Cell Death and Survival** | anoikis of prostate cancer cells | XIAP |
| | survival of pancreatic cancer cells | XIAP |
| | apoptosis of insect cell lines | XIAP |
| | survival of carcinoma cells | XIAP |
| | degeneration of retinal ganglion cells | XIAP |
| | apoptosis of ovarian surface epithelial cells | XIAP |
| | apoptosis of pancreatic cancer cells | XIAP |
| | loss of neurons | CAST,XIAP |
| | apoptosis of brain cells | CAST,XIAP |
| | apoptosis of endometrial cancer cell lines | XIAP |
| | apoptosis of renal tubule | TFAP2B |
| | apoptosis of neurons | CAST,PPP2R2B,XIAP |
| | apoptosis of carcinoma cell lines | TFAP2B,XIAP |
| | apoptosis of lung cancer cell lines | TFAP2B,XIAP |
| | cell death of neuroblastoma cell lines | CAST,DST |
| | anoikis of intestinal cell lines | XIAP |
| | apoptosis of leukocytes | CAST,PPP2R2B,XIAP |
| | apoptosis of T-cell hybrid cells | XIAP |
| | apoptosis of granulosa cells | XIAP |
| **Cell Morphology** | coalignment of microfilaments | DST |
| | coalignment of neurofilaments | DST |
| | polarization of Schwann cells | DST |
| | binucleation of fibroblasts | PCNT |
| | morphology of filopodia | CAST |
| | polarization of neutrophils | CAST |
| | polarization of cells | CAST,DST |
| | cell spreading of blood platelets | CAST |
| | extension of lamellipodia | CAST |
| | morphology of cellular protrusions | CAST,DST |
| | abnormal morphology of myelin sheath | DST |
| | formation of autophagosomes | CAST |

| | | |
|---|---|---|
| **Cell-To-Cell Signaling and Interaction** | dissociation of focal adhesions | CAST |
| | activation of ova | CAST |
| | modulation of dopamine | HTR2C |
| | activation of dopaminergic neurons | HTR2C |
| | cochlear microphonics | TECTA |
| | concentration of dopamine | HTR2C,XIAP |
| | compound action potential of cochlear nerve | TECTA |
| | secretion of acetylcholine | HTR2C |
| | synaptic transmission of superior cervical ganglion neurons | CAST |
| **Cellular Assembly and Organization** | formation of phagosomes | CAST,CDC27 |
| | coalignment of microfilaments | DST |
| | coalignment of neurofilaments | DST |
| | dissociation of focal adhesions | CAST |
| | formation of annulate lamellae | NUP153 |
| | transport of axons | DST |
| | secretion of alpha granules | CAST |
| | organization of cytoplasmic microtubule | DST |
| | extension of lamellipodia | CAST |
| | shortening of telomeres | TFAP2B |
| | nucleation of microtubules | PCNT |
| | organization of mitotic spindle | PCNT |
| | disruption of cytoskeleton | CAST |
| | formation of autophagosomes | CAST |
| **Cellular Compromise** | binucleation of fibroblasts | PCNT |
| | neurodegeneration of CA1 neuron | XIAP |
| | breakdown of nuclear envelope | NUP153 |
| | endoplasmic reticulum stress response of neuroblastoma cell lines | DST |
| | degeneration of retinal ganglion cells | XIAP |
| | disorganization of cytoskeleton | DST |
| | fragmentation of mitochondria | PPP2R2B |
| | fragmentation of Golgi apparatus | DST |
| | disruption of cytoskeleton | CAST |
| **Cellular Development** | osteoclastogenesis of macrophage cancer cell lines | CAST |

| | | |
|---|---|---|
| | osteoclastogenesis of bone-marrow-derived monocyte/macrophage precursor cells | CAST |
| | differentiation of adipoblasts | CAST |
| **Cellular Function and Maintenance** | formation of phagosomes | CAST,CDC27 |
| | function of gamma cells | MYT1 |
| | endoplasmic reticulum stress response of neuroblastoma cell lines | DST |
| | secretion of alpha granules | CAST |
| | function of outer hair cells | TECTA |
| | organization of cytoplasmic microtubule | DST |
| | extension of lamellipodia | CAST |
| | nucleation of microtubules | PCNT |
| | organization of mitotic spindle | PCNT |
| | axonal transport | DST |
| | formation of autophagosomes | CAST |
| **Cellular Movement** | transport of axons | DST |
| | chemokinesis of neutrophils | CAST |
| | migration of bladder cancer cell lines | XIAP |
| **Cellular Response to Therapeutics** | sensitivity of tumor cell lines | XIAP |
| **Connective Tissue Development and Function** | re-entry into cell cycle progression of fibroblast cell lines | CAST |
| | osteoclastogenesis of bone-marrow-derived monocyte/macrophage precursor cells | CAST |
| | differentiation of adipoblasts | CAST |
| **Connective Tissue Disorders** | microcephalic osteodysplastic primordial dwarfism, type 2 | PCNT |
| | tumorigenesis of fibroblast cell lines | CAST |
| **Dermatological Diseases and Conditions** | infection of epithelial cell lines | CAD,NUP153, XIAP |
| | malignant cutaneous melanoma cancer | CDC27,MLL3, TECTA |
| | blister | DST |
| **Developmental Disorder** | Char syndrome | TFAP2B |
| | microcephalic osteodysplastic primordial dwarfism, type 2 | PCNT |
| | Seckel syndrome 4 | PCNT |
| | postaxial polydactyly | TFAP2B |

| | | |
|---|---|---|
| | dystrophy of neurites | CAST |
| | congenital anomaly of musculoskeletal system | CAST,PCNT,TFAP2B |
| **DNA Replication, Recombination, and Repair** | organization of mitotic spindle | PCNT |
| **Drug Metabolism** | hydrolysis of D-methylphenidate | CES1 |
| | hydrolysis of cocaine | CES1 |
| | hydrolysis of irinotecan | CES1 |
| | modulation of dopamine | HTR2C |
| | concentration of dopamine | HTR2C,XIAP |
| **Embryonic Development** | ventralization of embryo | XIAP |
| | development of collecting tubule | TFAP2B |
| | osteoclastogenesis of bone-marrow-derived monocyte/macrophage precursor cells | CAST |
| **Endocrine System Development and Function** | release of corticosterone | HTR2C |
| | quantity of PTH in blood | TFAP2B |
| **Endocrine System Disorders** | hypocalciuria | TFAP2B |
| | hyperprolactinemia | HTR2C |
| | hypocalcemia | TFAP2B |
| **Gastrointestinal Disease** | primary sclerosing cholangitis | XIAP |
| **Hematological Disease** | hyperphosphatemia | TFAP2B |
| | childhood acute lymphoblastic leukemia | XIAP |
| | hypocalcemia | TFAP2B |
| **Hematological System Development and Function** | chemokinesis of neutrophils | CAST |
| | polarization of neutrophils | CAST |
| | osteoclastogenesis of bone-marrow-derived monocyte/macrophage precursor cells | CAST |
| | quantity of foam cells | CES1 |
| | cell spreading of blood platelets | CAST |
| **Hematopoiesis** | osteoclastogenesis of bone-marrow-derived monocyte/macrophage precursor cells | CAST |
| **Hepatic System Disease** | primary sclerosing cholangitis | XIAP |
| **Hereditary Disorder** | Char syndrome | TFAP2B |
| | X-linked familial hemophagocytic lymphohistiocytosis | XIAP |
| | dystonia mysculorum deformans 2 | DST |

| | | |
|---|---|---|
| | microcephalic osteodysplastic primordial dwarfism, type 2 | PCNT |
| | spinocerebellar ataxia 12 | PPP2R2B |
| | spinocerebellar ataxia recessive 9 | ADCK3 |
| | Seckel syndrome 4 | PCNT |
| | X-linked lymphoproliferative syndrome | XIAP |
| | childhood onset deafness DFNB8 | TECTA |
| | spinocerebellar ataxia | ADCK3,PPP2R2B |
| | Schizophrenia | CAD,HTR2C, MLL3,NBPF15 (includes others) |
| | hebephrenic schizophrenia | HTR2C |
| | paranoid schizophrenia | HTR2C |
| | X-linked hereditary disease | CAST,XIAP |
| **Immune Cell Trafficking** | chemokinesis of neutrophils | CAST |
| **Immunological Disease** | X-linked familial hemophagocytic lymphohistiocytosis | XIAP |
| **Immunological Disease** | X-linked lymphoproliferative syndrome | XIAP |
| | seasonal allergic rhinitis | HTR2C |
| | allergic rhinoconjunctivitis | HTR2C |
| | childhood acute lymphoblastic leukemia | XIAP |
| **Infectious Disease** | infection of embryonic cell lines | CAD,NUP153, XIAP |
| | infection of epithelial cell lines | CAD,NUP153, XIAP |
| | infection of kidney cell lines | CAD,NUP153, XIAP |
| | infection of cells | CAD,CLTCL1, NUP153,XIAP |
| **Inflammatory Disease** | seasonal allergic rhinitis | HTR2C |
| | allergic rhinoconjunctivitis | HTR2C |
| | primary sclerosing cholangitis | XIAP |
| **Inflammatory Response** | chemokinesis of neutrophils | CAST |
| | polarization of neutrophils | CAST |
| | quantity of foam cells | CES1 |
| | cell spreading of blood platelets | CAST |
| **Lipid Metabolism** | elimination of cholesterol | CES1 |
| | synthesis of D-erythro-C16-ceramide | XIAP |
| | release of corticosterone | HTR2C |
| | secretion of GABA | HTR2C |

| | | |
|---|---|---|
| | concentration of bile acid | MLL3 |
| **Lymphoid Tissue Structure and Development** | osteoclastogenesis of bone-marrow-derived monocyte/macrophage precursor cells | CAST |
| **Metabolic Disease** | spinocerebellar ataxia recessive 9 | ADCK3 |
| | beta amyloidosis | CAST |
| **Molecular Transport** | elimination of cholesterol | CES1 |
| | concentration of dopamine | HTR2C,XIAP |
| | secretion of acetylcholine | HTR2C |
| | release of corticosterone | HTR2C |
| | secretion of GABA | HTR2C |
| | quantity of PTH in blood | TFAP2B |
| | concentration of bile acid | MLL3 |
| **Nervous System Development and Function** | coalignment of neurofilaments | DST |
| | activation of dopaminergic neurons | HTR2C |
| | cochlear microphonics | TECTA |
| | transport of axons | DST |
| | mechanotransduction of hair cells | TECTA |
| | compound action potential of cochlear nerve | TECTA |
| | function of outer hair cells | TECTA |
| | synaptic transmission of superior cervical ganglion neurons | CAST |
| | loss of neurons | CAST,XIAP |
| | abnormal morphology of myelin sheath | DST |
| | axonal transport | DST |
| **Neurological Disease** | dysmyelination of peripheral nervous system | DST |
| | dystonia mysculorum deformans 2 | DST |
| | microcephalic osteodysplastic primordial dwarfism, type 2 | PCNT |
| | spinocerebellar ataxia 12 | PPP2R2B |
| | spinocerebellar ataxia recessive 9 | ADCK3 |
| | childhood onset deafness DFNB8 | TECTA |
| | neurodegeneration of CA1 neuron | XIAP |
| | spinocerebellar ataxia | ADCK3,PPP2R2B |
| | onset of amyotrophic lateral sclerosis | XIAP |
| | suicidal behavior | CAD |

| | | |
|---|---|---|
| | beta amyloidosis | CAST |
| | microgliosis of brain | CAST |
| | dystrophy of neurites | CAST |
| | Schizophrenia | CAD,HTR2C, MLL3,NBPF15 (includes others) |
| | muscle spasm | TFAP2B |
| | tic disorder | HTR2C |
| | amyotrophic lateral sclerosis | HTR2C,XIAP |
| | Asperger syndrome | HTR2C |
| | tonic seizure | HTR2C |
| | bipolar II disorder | HTR2C |
| | cannabis dependence | HTR2C |
| | hebephrenic schizophrenia | HTR2C |
| | major depression | CAD,HTR2C |
| | paranoid schizophrenia | HTR2C |
| | vascular dementia | HTR2C |
| | anorexia | HTR2C |
| | psychomotor agitation | HTR2C |
| **Nutritional Disease** | anorexia | HTR2C |
| | adult-onset obesity | HTR2C |
| **Ophthalmic Disease** | allergic rhinoconjunctivitis | HTR2C |
| **Organ Development** | development of collecting tubule | TFAP2B |
| | osteoclastogenesis of bone-marrow-derived monocyte/macrophage precursor cells | CAST |
| **Organ Morphology** | sensitivity of basilar membrane | TECTA |
| | abnormal morphology of otolithic membrane | TECTA |
| | abnormal morphology of enlarged otoliths | TECTA |
| | abnormal morphology of dilated proximal convoluted tubule | TFAP2B |
| | abnormal morphology of tectorial membrane | TECTA |
| | opening of eyelid | MLL3 |
| **Organismal Development** | ventralization of embryo | XIAP |
| | development of collecting tubule | TFAP2B |
| | osteoclastogenesis of bone-marrow-derived monocyte/macrophage precursor cells | CAST |

| | | |
|---|---|---|
| **Organismal Injury and Abnormalities** | neurodegeneration of CA1 neuron | XIAP |
| | microgliosis of brain | CAST |
| | infection of embryonic cell lines | CAD,NUP153, XIAP |
| | hot flashes | HTR2C |
| | hemopericardium | XIAP |
| | size of lesion | CES1,XIAP |
| **Post-Translational Modification** | ubiquitination of protein | CAST,XIAP |
| **Protein Synthesis** | quantity of PTH in blood | TFAP2B |
| **Protein Trafficking** | targeting of green fluorescent protein | PPP2R2B |
| **Psychological Disorders** | suicidal behavior | CAD |
| | Schizophrenia | CAD,HTR2C, MLL3,NBPF15 |
| | Asperger syndrome | HTR2C |
| | bipolar II disorder | HTR2C |
| | cannabis dependence | HTR2C |
| | hebephrenic schizophrenia | HTR2C |
| | major depression | CAD,HTR2C |
| | paranoid schizophrenia | HTR2C |
| | vascular dementia | HTR2C |
| | anorexia | HTR2C |
| **Renal and Urological Disease** | infection of kidney cell lines | CAD,NUP153, XIAP |
| **Renal and Urological System Development and Function** | development of collecting tubule | TFAP2B |
| | abnormal morphology of dilated proximal convoluted tubule | TFAP2B |
| **Reproductive System Development and Function** | activation of ova | CAST |
| **Reproductive System Disease** | hyperprolactinemia | HTR2C |
| **Respiratory Disease** | growth of lung tumor | XIAP |
| | seasonal allergic rhinitis | HTR2C |
| | allergic rhinoconjunctivitis | HTR2C |
| **Skeletal and Muscular Disorders** | microcephalic osteodysplastic primordial dwarfism, type 2 | PCNT |
| | postaxial polydactyly | TFAP2B |
| | muscle spasm | TFAP2B |
| | congenital anomaly of musculoskeletal system | CAST,PCNT,TFAP2B |
| | psychomotor agitation | HTR2C |

| | | |
|---|---|---|
| | alveolar rhabdomyosarcoma | TFAP2B |
| **Skeletal and Muscular System Development and Function** | osteoclastogenesis of macrophage cancer cell lines | CAST |
| | osteoclastogenesis of bone-marrow-derived monocyte/macrophage precursor cells | CAST |
| **Small Molecule Biochemistry** | biosynthesis of pyrimidine | CAD |
| | elimination of cholesterol | CES1 |
| | hydrolysis of irinotecan | CES1 |
| | modulation of dopamine | HTR2C |
| | hydrolysis of inositol phosphate | HTR2C |
| | concentration of dopamine | HTR2C,XIAP |
| | synthesis of D-erythro-C16-ceramide | XIAP |
| | secretion of acetylcholine | HTR2C |
| | regulation of D-glucose | HTR2C |
| | release of corticosterone | HTR2C |
| | secretion of GABA | HTR2C |
| | quantity of PTH in blood | TFAP2B |
| | concentration of bile acid | MLL3 |
| **Tissue Development** | dissociation of focal adhesions | CAST |
| | development of collecting tubule | TFAP2B |
| | osteoclastogenesis of bone-marrow-derived monocyte/macrophage precursor cells | CAST |
| **Tissue Morphology** | quantity of foam cells | CES1 |
| | size of lesion | CES1,XIAP |
| | abnormal morphology of myelin sheath | DST |
| **Tumor Morphology** | anoikis of prostate cancer cells | XIAP |
| | survival of pancreatic cancer cells | XIAP |
| | survival of carcinoma cells | XIAP |
| | apoptosis of pancreatic cancer cells | XIAP |
| **Visual System Development and Function** | opening of eyelid | MLL3 |
